# Tc-99m Labeled HMPAO white Blood Cell Scintigraphy in Pediatric Patients

**DOI:** 10.4274/Mirt.165

**Published:** 2012-04-01

**Authors:** Funda Aydın, Arzu Kın Cengiz, Fırat Güngör

**Affiliations:** 1 Akdeniz University Medical School, Department of Nuclear Medicine, Antalya, Turkey; 2 Adnan Menderes University Medical School, Department of Nuclear Medicine, Aydın, Turkey

**Keywords:** 99mTc-HMPAO, scintigraphy, osteomyelitis, fever of unknown origin, appendicitis, Pediatrics

## Abstract

**Objective:** ^99m^Tc labeled hexamethylpropylene amine oxime (HMPAO) white blood cell (WBC) scintigraphy is a frequently used option for acute infection, particularly in pediatric patients. This scintigraphy is applied to detect sites of infection/inflammation in patients with fever of unknown origin, to find and follow up osteomyelitis, and to detect suspicion of acute appendicitis. The aim of this retrospective study was to evaluate the value of ^99m^Tc-HMPAO labeled WBC scintigraphy in pediatric patients.

**Material and Methods:** The study was conducted between January 2006 and December 2008 and included 13 patients (5 boys, 8 girls; mean age 6.9±6.2 years). Those patients who had suspicion of bone infection (n=7), fever of unknown origin (n=3), and suspicion of acute appendicitis (n=3) were evaluated retrospectively. ^99m^Tc-HMPAO labeled WBC scintigraphy imaging was performed to all patients. Diagnosis was done according to operation and pathological results or clinical follow-up.

**Results:** ^99m^Tc-HMPAO labeled WBC scintigraphy has been found to be true positive in 6 cases, true negative in 6 cases, and false negative in one patient who had fewer unknown origin. The false negative case has been found to have encephalitis with MRI.

**Conclusion:** Leukocyte scintigraphy has been described as a useful diagnostic tool in the diagnosis of suspicion of bone infection, fever of unknown origin and suspicion of acute appendicitis. ^99m^Tc-HMPAO labeled WBC scintigraphy is a rapid and very accurate method for detecting those pathologies. Our results showed that WBC scintigraphy might be reliably used for diagnosis of suspected bone infection and acute appendicitis, fever of unknown origin, and acute appendicitis, in pediatric patient population.

**Conflict of interest:**None declared.

## INTRODUCTION

The early and accurate localization of infectious foci is a major challenge in contemporary nuclear medicine. Early and accurate diagnosis and localization allow prompt and successful treatment and decrease associated morbidity (1).

Clinicians have tried to localize, uncover, and monitor sources of infections using various diagnostic schemes including clinical examination and laboratory tests, as well as biologic and anatomic imaging (2). 

Nuclear medicine plays an important role in the evaluation of patients (pediatric and adult) suspected of harboring infection. Although Tc-99m MDP, Ga-67 citrate and F-18 FDG all are useful, in vitro labeled leukocyte imaging is the radionuclide gold standard for imaging most infections (3). 

Imaging with the use of ex vivo-labeled autologous leukocytes with Indium-111 (^111^In) was developed in the 1970s (4,5). The approach is to use a neutral, lipophilic chelate of ^111^In which is able to enter cells by passive diffusion followed by binding to intracellular macromolecules (6). 

The clinical value of early studies with ^111^In-labeled leukocytes led to the search for an analogous method using Technetium-99m (^99m^Tc), which is logistically easier to use because of its routine availability from an on-site Molybdenum-99 (^99^Mo)/ ^99m^Tc generator (6). 

Since 1986, ^99m^Tc hexamethylpropylene amine oxime labeled white blood cell (^99m^Tc-HMPAO labeled WBC) scintigraphy has been a frequently used option for acute infection, particularly in pediatric patients (1,3,7,8,9). Conceptually it is similar to the ^111^In-oxine approach in that a neutral, lipophilic chelate enters the cells by passive diffusion and is changed into a chemical form which is not able to exit across the cell membrane (6). 

The principal clinical indications for using 99mTc-HMPAO labeled WBC scintigraphy are inflammatory bowel disease, acute osteomyelitis and to a lesser extent, occult fever (10). This imaging is also indicated for acute soft tissue infection and to detect suspicion of acute appendicitis (11). WBC scintigraphy is useful in the pediatric group, where ^99m^Tc-methylene diphosphonate (MDP) has low specificity (12).

The aim of this retrospective study was to evaluate the value of ^99m^Tc-HMPAO labeled WBC scintigraphy for the diagnosis of infection and inflammation in pediatric patients.

## MATERIALS AND METHODS

**Patient Population**

This study was designed as a retrospective clinical trial. Thirteen patients (5 boys and 8 girls; aged between 1-17 years; mean age 6.9±6.2 years) were included in this study from January 2006 and December 2008. Patients were referred for ^99m^Tc-HMPAO labeled WBC scintigraphy to evaluate for infection suspected in bone (n=7.54%), fever of unknown origin (FUO) (n=3.23%), and suspicion of acute appendicitis (n=3.23%) (Table 1). To discriminate bone infection from soft-tissue infection, we used a combination of labeled leukocyte scintigraphy and ^99m^Tc MDP three-phase bone scanning. 

Before scintigraphy, the children’s parents had to sign a written informed consent where all information about the leukocyte labeling and imaging were explained. 

**Labeling of WBCs**

Leukocytes were labeled in vitro with ^99m^Tc-HMPAO (Ceretec, Amersham Healthcare) using a consensus protocol that was described earlier (13).

**Imaging Protocol**

Planar whole-body sweep and static imaging were typically performed under a gamma camera (Toshiba GCA 501 S or Sopha DST-XLi) equipped with low-energy all purpose or high resolution collimator starting at the 30^th^ min following the injection of 37-74 MBq ^99m^Tc-HMPAO labeled WBC. Patients were instructed to void before imaging. Imaging was repeated at 1^th^, 2^th^, and 4^th^ h. All images were acquired for 10 minutes duration. 

**Image Interpretation**

The scintigraphic images were reviewed by two nuclear medicine specialists making a comparison between the acquisitions at various times by the following evaluation criteria:

(1) When the scans showed significant change in leukocyte distribution between 1^th^ hour images compared to the 4th hour images, they were considered positive.

(2) The presence of areas of progressive leukocyte concentration in proportion to time, was also considered as sign of presence of disease.

(3) Scintigraphic examination not matching the above mentioned criteria were considered as negative. Only the scans judged as positive or negative by both specialists were finally accepted as being so. Diagnosis was done according to operation and pathological results, or clinical follow-up.

**Analysis of Scintigrams**

^99m^Tc-HMPAO labeled WBC scintigraphy was considered true-positive when an infectious focus identified by scintigraphy was later verified by another method (surgery, biopsy, other imaging modalities, and clinical follow-up). Scintigrams identifying foci that were never verified during follow-up or foci that were verified as noninfectious were considered false-positive. A scintigram was considered true-negative when no infectious process was found during follow-up. Scintigrams were considered false-negative if an infectious focus was identified by other methods. 

## RESULTS

Of the 13 patients studied, a final result was obtained in 12 (92%). ^99m^Tc-HMPAO labeled WBC scintigraphy was found to be true positive in 6 of 13 patients, true negative results were obtained for 6 cases, and false negative in one patient who had FUO. The false negative case has been found encephalitis with MRI (Table 1). 

**Figure 1:**
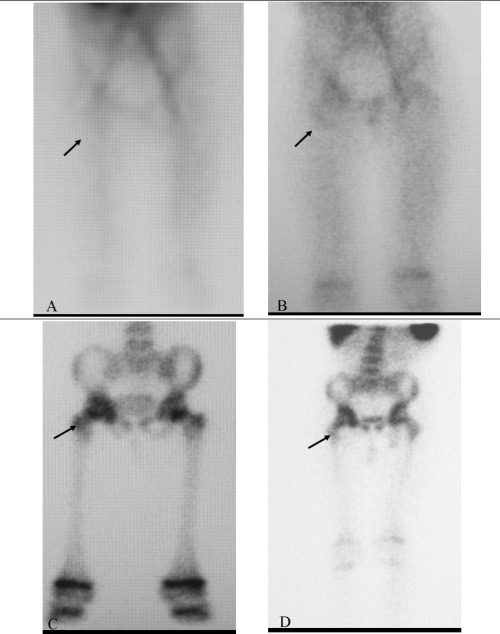
5 year old female patient with suspected right hip infection.Figure 1A, Figure 1B, Figure 1C. Three-phase bone scintigraphy shows positiveuptake in the region of right hip (arrow). Figure 1D. ^99m^Tc-HMPAOlabeled WBC scintigraphy shows positive uptake in the same region (arrow)compatible with osteomyelitis. Result: Infected right hip

Three-phase bone scintigraphy was positive in all children with the suspicion of acute bone infection in our series. ^99m^Tc-HMPAO labeled WBC scintigraphy was found to be true positive in 5 of 7 patients who were evaluated for infection suspected in bone (Figure 1). These cases were treated with antibiotics for more than 6 weeks. Four patients true negative for osteomyelitis did not receive antibiotics.

One of 3 cases who had suspicion of acute appendicitis was operated, and the final diagnosis was acute appendicitis. The other 2 patients had negative scintigraphy for acute appendicitis, and they were not operated on. These patients showed clinical improvement without any treatment.

^99m^Tc-HMPAO labeled WBC scintigraphy was negative in 3 patients who were considered as FUO. Two of them were true negative because no cause was found, and they spontaneously recovered and no fever was observed at the end of follow-up. The remaining 1 patient, has been found to have encephalitis and abscess in left frontal area with MRI (WBC scintigraphy was false negative). 

^99m^Tc HMPAO labeled WBC scintigraphy yielded no false positive results.

## DISCUSSION

^99m^Tc-HMPAO labeled WBCs normally accumulate in the liver, spleen, bone marrow, kidneys, and gastrointestinal tract. The principal clinical indications for using ^99m^Tc-HMPAO labeled WBC scintigraphy are inflammatory bowel disease (IBD), acute osteomyelitis and to lesser extent, occult fever (10). ^99m^Tc-HMPAO labeled WBC imaging is also indicated for acute soft-tissue and abdominal sepsis (11). In children and where the resolution is bad, such as for small-bowel involvement in Chron’s disease, 99mTc-HMPAO labeled WBC scintigraphy is preferred because of superior resolution, higher count density and lower radiation dose (14).

Three-phase bone scintigraphy with ^99m^Tc-MDP is the study of choice for diagnosing osteomyelitis in bones, and to discriminate bone infection from soft-tissue infection, but this is less specific in patients with fractures or prosthesis (15,16). ^99m^Tc-leukocytes are generally more sensitive for detection of acute osteomyelitis than of chronic osteomyelitis. They are also useful in the pediatric group, where ^99m^Tc-MDP has low specificity (12). It is well known that a diagnosis can be reached without resorting to this diagnostic method in the majority of patients with non-violated bone tissue. These cases are hematogenous osteomyelitis which can be easily detected by clinical signs and symptoms, laboratory data and three-phase bone scintigraphy (17). Therefore, with a few exceptions, the need for WBCs is restricted to osteomyelitis that has developed in bone already affected by structural changes (previous diseases, orthopedic devices, prostheses etc.) or suspected of having been caused by the spread to the bone of contiguous foci of infection (exposed fractures, osteomyelitis underlying skin ulcers in diabetic foot, etc.) (17). Three-phase bone scintigraphy was positive in all children with the suspicion of bone infection in our series. However, ^99m^Tc- HMPAO labeled WBC scintigraphy was found to be positive in 5 patients and these cases were true-positive, and there were no false-positive or false-negative results in our group of patients with the suspicion of bone infection.

FUO is an illness of at least 3 weeks duration, with several episodes of fever exceeding 38.3°C, and no diagnosis after an appropriate inpatient or outpatient evaluation. The underlying causes of FUO are numerous; the most common causes are infections, malignancies and collagen vascular disease (18,19). Identifying the source of an FUO is often difficult; however, radionuclide studies can provide important information. Several investigators have reported that labeled leukocyte imaging is a useful investigation method for this indication (20,21,22). Recently Seshadri et al reported on labeled leukocyte imaging in 54 patients with FUO. Although the sensitivity of the test was only 60% and the specificity was 73%, the authors found that a negative study in patients with spontaneous FUO virtually excludes infection/ inflammation, and concluded that labeled leukocyte imaging is useful in this population (23). 

In this study, 99mTc-HMPAO labeled WBC scintigraphy was negative in 3 patients who were considered to have FUO. Two of them were true negative because no cause was found, and they spontaneously recovered and no fever was observed at the end of the follow-up. The remaining 1 patient has been found to have encephalitis with MRI (WBC scintigraphy was false negative). The application of WBC scintigraphy in brain abscess diagnosis has been reported sporadically and described in greater detail in two published studies (24,25). Rehncrona et al. (24) observed 111In uptake in four of five brain abscess imaged 24 and 48 h post injection. Bellotti et al. (25) claimed 100% sensitivity and 94% specificity of this method for brain abscess detection. However, there are some obvious pitfalls and shortcomings. Cerebral abscess in patients on high-dose steroid treatment may not be detected by WBC scintigraphy (26). Quartey et al. (27) found in rabbit experiments that dexamethasone impedes bacterial killing in brain abscess in animals on antibiotic treatment. This was accompanied by sparse infiltration by granulocytes and compromised granulation tissue and fibrous capsule formation. Neuwelt et al. (28) found high- and medium-dose dexamethasone to suppress macrophage and glial response and to decrease collagen formation. Our false negative case was using dexamethasone and antibiotic treatment. 

^18^F-FDG PET/CT is a useful imaging tool in patients with FUO. When systemic diseases are excluded by other diagnostic tests, a negative PET/CT may avoid the need for further investigation. The synergy of combined anatomic-metabolic information is of incremental value in the diagnostic work-up of FUO. A few studies have found that ^18^F-FDG PET and PET/CT might be useful to detect the inflammatory focus in FUO (29,30).

Diagnosing acute appendicitis in children with equivocal signs and symptoms is usually difficult. The usual approach to the patient is hospital observation and frequent reexamination. 

However, many surgeons are reluctant to delay surgery because of the risk of perforation and a negative laparatomy (31). The rate of complications, including death, is directly correlated with delay in diagnosis and surgery (32). The fact that no single laboratory test is 100% accurate for diagnosing appendicitis exacerbates the problem. Plain radiography, barium enema, graded compression ultrasonography and computed tomography have been employed in the attempt to diagnose acute appendicitis with atypical presentations. Since these tests all have limitations and drawbacks, their use has not reduced the number of negative laparotomies or made a significant impact on the clinical management of these patients (33). When all these factors are considered, it is not surprising that attempts to diagnose patients with appendicitis can lead to frustration and confusion for both the patient and the doctor.

There have been a few studies on the use of ^99m^Tc-HMPAO WBC scintigraphy in pediatric cases of suspected appendicitis (34,35). Chang et al. (31) showed that ^99m^Tc-HMPAO labeled WBC scintigraphy is a useful and non-invasive test for confirming the clinical diagnosis of suspected acute appendicitis in children. In this study, ^99m^Tc-HMPAO labeled WBC scintigraphy had a sensitivity of 96.7%, specificity of 80% and accuracy of 88% (31). However, Kanegaye et al. concluded that ^99m^Tc-HMPAO WBC scintigraphy is neither accurate nor reliable as a diagnostic test in pediatric patients with an initial clinical presentation equivocal for appendicitis. Their reported sensitivity and specificity rates for appendicitis using 99mTc-HMPAO labeled WBC scintigraphy are the lowest in the literature (35).

There were 3 cases with suspicion of acute appendicitis in our group of patients. One of 3 cases was operated because the scan was considered positive for acute appendicitis, and final diagnosis was acute appendicitis. The other 2 patients had negative scintigraphy for acute appendicitis, and they were not operated on. These patients showed clinical improvement without any treatment. 

The ^99m^Tc-HMPAO WBC scintigraphy as an alternative to the invasive gold standard represented by endoscopy with biopsy is a reliable method both for diagnosis and follow-up in pediatric inflammatory bowel disease (IBD) (36,37). Just a few studies about pediatric patients can be found in the literature, and the results of these studies showed sensitivity between 84% and 93%, specificity between 81% and 93% in the diagnosis of IBD (38,39). The ^99m^Tc-HMPAO labeled WBC scintigraphy has proved itself in correctly defining IBD remission and relapses and in defining lesions location, extension, and severity (37). In children with possible IBD the ^99m^Tc-HMPAO labeled WBC scintigraphy, when compared with endoscopy and biopsy, has a sensitivity rate of 93%, a specificity rate of 97% (38). These results suggest that ^99m^Tc-HMPAO labeled WBC scintigraphy is useful as an initial screening method to exclude IBD (38). Therefore, ^99m^Tc-HMPAO labeled WBC scintigraphy is a valid, minimally invasive, and reproducible technique which can give accurate information about the presence or absence of lesions and the intensity and extension of disease in patients affected by IBD in the active state, according to literature data (36,37,38,39).

In summary, leukocyte scintigraphy has been described as a useful diagnostic tool in the diagnosis of suspicion of bone infection, fever of unknown origin and suspicion of acute appendicitis. Tc-99m HMPAO labeled WBC scintigraphy is a rapid and very accurate method for detecting those pathologies.

Our results showed that WBC scintigraphy might be reliably used for diagnosis of suspected bone infection, acute appendicitis, and fever of unknown origin in pediatric patient population. 

Furthermore, larger, well-designed, prospective studies are needed to validate and to implement the strategy of using this imaging modality as an initial diagnostic investigation in pediatric patients.

## Figures and Tables

**Table 1:**
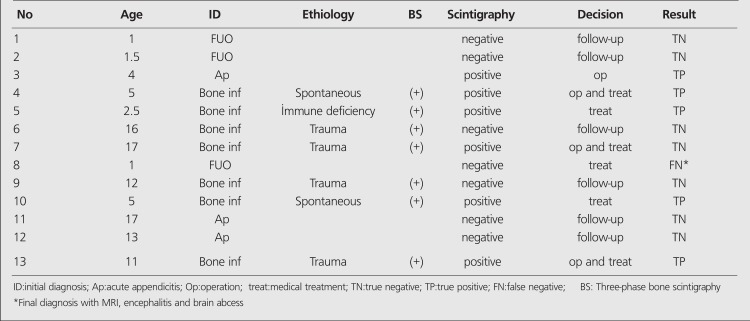
Summary of the patients
